# Task-shifting alcohol interventions for HIV+ persons in Kenya: a cost-benefit analysis

**DOI:** 10.1186/s12913-017-2169-4

**Published:** 2017-03-28

**Authors:** Omar Galárraga, Burke Gao, Benson N. Gakinya, Debra A. Klein, Richard G. Wamai, John E. Sidle, Rebecca K. Papas

**Affiliations:** 1 Brown University School of Public Health, G-S121-7, 121 South Main Street, Providence, RI 02912 USA; 2 Brown University Alpert Medical School, 222 Richmond Street, Providence, RI 02912 USA; 3 Moi University & Moi Teaching and Referral Hospital, Nandi Rd, Eldoret, Kenya; 4 Edify Youth, St Paul, MN USA; 5 Northeastern University, Integrated Initiative for Global Health, 360 Huntington Avenue, 220G RP, Boston, MA 02115 USA; 6 Indiana University School of Medicine, Indianapolis, IN USA; 7 Department of Psychiatry and Human Behavior, Brown University Alpert Medical School, Providence, RI 02912 USA

**Keywords:** Task-shifting, Alcohol, Cognitive-behavioral-therapy, CBT, HIV, AIDS, Cost-benefit-analysis, Kenya, Sub-Saharan Africa

## Abstract

**Background:**

Among HIV+ patients, alcohol use is a highly prevalent risk factor for both HIV transmission and poor adherence to HIV treatment. The large-scale implementation of effective interventions for treating alcohol problems remains a challenge in low-income countries with generalized HIV epidemics. It is essential to consider an intervention’s cost-effectiveness in dollars-per-health-outcome, and the long-term economic impact —or “return on investment” in monetary terms.

**Methods:**

We conducted a cost-benefit analysis, measuring economic return on investment, of a task-shifted cognitive-behavioral therapy (CBT) intervention delivered by paraprofessionals to reduce alcohol use in a modeled cohort of 13,440 outpatients in Kenya. In our base-case, we estimated the costs and economic benefits from a societal perspective across a six-year time horizon, with a 3% annual discount rate. Costs included all costs associated with training and administering task-shifted CBT therapy. Benefits included the economic impact of lowered HIV incidence as well as the improvements in household and labor-force productivity. We conducted univariate and multivariate probabilistic sensitivity analyses to test the robustness of our results.

**Results:**

Under the base case, total costs for CBT rollout was $554,000, the value of benefits were $628,000, and the benefit-to-cost ratio was 1.13. Sensitivity analyses showed that under most assumptions, the benefit-to-cost ratio remained above unity indicating that the intervention was cost-saving (i.e., had positive return on investment). The duration of the treatment effect most effected the results in sensitivity analyses.

**Conclusions:**

CBT can be effectively and economically task-shifted to paraprofessionals in Kenya. The intervention can generate not only reductions in morbidity and mortality, but also economic savings for the health system in the medium and long term. The findings have implications for other countries with generalized HIV epidemics, high prevalence of alcohol consumption, and shortages of mental health professionals.

**Trial registration:**

This paper uses data derived from “*Cognitive Behavioral Treatment to Reduce Alcohol Use Among HIV-Infected Kenyans (KHBS)*” with ClinicalTrials.gov registration NCT00792519 on 11/17/2008; and preliminary data from “*A Stage 2 Cognitive-behavioral Trial: Reduce Alcohol First in Kenya Intervention*” (NCT01503255, registered on 12/16/2011).

## Background

Alcohol use is responsible for 13.5% of global deaths due to infectious diseases including HIV, and 5.1% of disability-adjusted life years (DALYs) [1, 2]. The high worldwide alcohol use related morbidity and mortality suggests an urgent need for a global focus on treatment of alcohol use disorders [3–5]. Alcohol consumption negatively impacts the effectiveness of prevention efforts by facilitating HIV transmission through increased sexual risk behavior as well as HIV treatment non-adherence [6–11]. Although dramatic progress against HIV/AIDS has been made in sub-Saharan Africa [12] –with disease incidence decreasing by 25% in 22 countries from 1990 to 2009 and treatment access expanding from 50,000 to over 5 million persons from 2002 to 2012 [13] – the progress towards “zero new infections and zero AIDS-related deaths” promoted by UNAIDS [14] is hampered by several challenges. These include the growing HIV prevalence due to expanded lifespans, limited accessibility to antiretrovirals (ARVs), and continued suboptimal adherence to treatment [13, 14]. While increased access to ARVs improves overall health by lowering viral load and acting as a protective factor [15, 16], the predictive ability of adherence [17, 18] is strongly mediated by alcohol use [8, 19–23]. This suggests that alcohol use can present a significant barrier to the *Zero Campaign* by increasing HIV infectivity. The evidence suggests a linear dose-response relationship between alcohol use and risk for comorbid and AIDS-defining illnesses [24]. That is, although high levels of alcohol use are associated with higher rates of AIDS-related complications and higher health care costs, *any* level of alcohol use places a patient at higher risk for such complications and higher costs—thus suggesting that there is no “safe” level of alcohol consumption for HIV-infected patients. For this reason, we consider alcohol use of all levels as harmful for HIV+ persons throughout this paper.

In sub-Saharan Africa, Kenya is among countries with high and hazardous alcohol consumption, 22.7% of adults (age 15+) using alcohol within the past 12 months, and an estimated 3.2% of adults (age 15+) exhibiting alcohol use disorders [2]. Furthermore in a survey among school-going Kenyan youth, 48.9% have drunk alcohol [25]. Because harmful alcohol use behavior is classified as a mental health disorder, known as alcohol use disorder (or AUD), and because such behavior can be associated with spread of HIV [9, 26–28], it is important that those with AUD receive appropriate mental health treatment. Unfortunately, throughout much of the world, there exists a large treatment gap between psychiatric service availability and the high burden of mental disorders including substance abuse [29]. In Kenya in particular, the Ministry of Health reports that 20–30% outpatient visits are for mental disorders [30], and only 15% of mental health patients receive treatment [31]. Furthermore, despite an estimated 25% of patients in general health clinics suffering from alcohol and substance abuse disorders, only 0.1% of patients in general health clinics had their alcohol abuse problems picked up by clinicians [31]. This is likely because of the dearth of mental health care workers. In 2010, Kenya employed approximately 75 psychiatrists, only 12 of whom work across the eight provincial and 250 district hospital system – one psychiatrist for every province of 3–5 million people [32]. There were 250 trained psychiatric nurses deployed in psychiatry in Kenya, so approximately one psychiatric nurse per 160,000 Kenyans [32]. In the current context of acute shortages of specialist health workers, high alcohol use, and high HIV prevalence [33–35], there are calls for innovative cost-effective strategies to reduce alcohol use in sub-Saharan Africa, and in Kenya in particular [36, 37].

One promising model to accelerate delivery is task-shifting of services in which tasks performed by professionals are delegated to those with less formal education or training called paraprofessionals [38–41]. Shifting of mental health services through training additional personnel could be critical to meet demand [32, 42]. Task-shifting to scale-up other HIV prevention interventions in Kenya offers a practical model for healthcare delivery [43]. Task-shifting HIV and alcohol interventions may promote behavior change among larger numbers of people living with HIV, including men who generally utilize less HIV testing, less therapy and display lower adherence to care [44–46].

Given the limited number of mental health professionals in Kenya, we developed a cognitive behavioral therapy (CBT) to reduce alcohol consumption, and a framework of paraprofessional training and supervision to task-shift this CBT. CBT has been shown to positively influence health behavior by engaging participants in a deliberate exploration of thoughts, actions and feelings by which participants learn coping skills to handle high-risk substance use situations [47]. CBT was selected for the Kenyan adaptation because of its strong empirical support in both individual and group formats to reduce substance abuse [48, 49], durability of treatment effects, and prior successful applications in sub-Saharan Africa to reduce risky sexual behaviors among HIV-infected Zambian couples [50] and to improve mood among Nigerian surgical patients [51]. Furthermore, because of its highly-structured format, CBT was feasible for training paraprofessionals and for delivery to those with limited formal education. On the contrary, using Medication Assisted Therapy (MAT) was not feasible in this resource-limited setting due to extremely limited health care professionals to deliver or monitor the medication. Additionally, MAT typically requires longer delivery to curb alcohol use, recommended from 4 months [52] up to 12 months or longer [53].

In a previous pilot study, we found task-shifted CBT to be effective at reducing alcohol use, with a reported alcohol abstinence rate of 69% at a 90 day follow-up (vs. 38% usual care) [47, 54]. As mentioned earlier, all levels of alcohol use—even low levels—have been shown to have a negative impact on HIV+ persons [24]. Therefore, our pilot study included any HIV+ patients who had any amount of alcohol in the previous month, as well as a score of 3 on AUDIT-C or endorsement of binge drinking on a monthly basis. Furthermore, the level of competency of this task-shifted CBT was independently rated to be equivalent to therapy delivered by college-educated therapists in the U.S. [47, 54].

The present economic evaluation study has three aims. First, we describe field-based costs of a feasible rollout of CBT to reduce alcohol use among 13,440 HIV-infected outpatients at 12 sites in Kenya. Our assumptions are based on two CBT trials in western Kenya [47, 54]. Second, we estimate the potential economic benefits of the rollout in medium- and long-term based on simulation methods. Third, we calculate the potential medium to long-term benefit-to-cost ratio (BCR).

We chose to conduct a cost-benefit analysis for two reasons. First, previous research suggests that CBT may be cost-effective in the Kenyan context, but cost-saving only under very strict assumptions [37]. We hypothesized that including the additional potential economic impact of the CBT intervention in the Kenyan setting would provide a more comprehensive assessment. Thus, cost-benefit analysis (comparing both costs and all benefits in monetary terms) would be better suited for illustrating this point than cost-effectiveness analysis (which compares costs in monetary terms, and effects given only as health outcomes) or cost-utility analysis (which uses cost per utility effect such as dollar per quality-adjusted life year) [55, 56]. To show that CBT is effective and cost-saving is not a trivial task because only a few interventions are highly-effective and also save costs [57]. Second, modelling based on effectiveness outcomes alone has already been completed using our CBT estimates [36], making cost-benefit analysis (that includes monetary evaluation of health and economic benefits) the next logical step in this line of research. Our cost-benefit analysis offers useful novel information regarding the health and economic benefits of CBT.

## Methods

A cost-benefit analysis is an economic evaluation technique which places a monetary valuation on a health program’s costs and benefits, allowing for the comparison of a health program’s incremental cost to its incremental benefits in corresponding monetary units [58]. The model presented in this paper estimates the costs and benefits of rolling out a task-shifted CBT intervention to reduce alcohol use among persons living with HIV in Kenya from a societal perspective, including beneficial economic impact over and above reductions in mortality and morbidity. Benefits included those associated with lowered HIV incidence and improvements in household as well as labor force productivity (Table 1). Costs included all costs associated with training and administering task-shifted CBT (Tables 2 and 3). There is inherent uncertainty when placing a monetary valuation on a program’s potential future costs and benefits. To help account for this uncertainty we created sensitivity analyses which vary our model’s key assumptions across different ranges as described in the Sensitivity analyses section of this paper.

**Table 1:** Input parameters to model costs and benefits (per CBT participant)

Parameter	Value	Range	Sources/Reference
Benefit 1: Decreased incidence of HIV	$41	$39 to $43	See Table 2 and Table 3
Efficacy of alcohol intervention:			
Percentage of patients reporting abstinence at 90 day follow-up (CBT intervention)	69%		[54]
Percentage of patients reporting abstinence at 90 day follow-up (Usual Care)	38%		[54]
Difference between intervention and usual care in percentage of patients reporting abstinence at 90 day follow-up (parameter 1.1)	31%	21% to 41%	
Percentage of HIV incidence attributable to alcohol consumption (parameter 1.2)	13%	1.8 to 16.5%	[86]
Consumer Price Index/Inflation Rates:			
Average 2009 Kenyan CPI	100		[60]
Average 2010 Kenyan CPI	106.265		
Average 2011 Kenyan CPI	121.17		
Average 2013 Kenyan CPI	140.103		
Costs of treating new case of HIV:			
Average non-drug related costs (2009 USD)			
Lab tests	32	29.2 to 36.2	[66]
Visits	24	18.8 to 29.2	
Support services	0.4	0.10 to 0.90	
Fixed costs	32	22.4 to 46.6	
Sum of mean non-drug related costs (2013 USD)	124		[66]
Median drug costs in USD:			
12 Month tenofovir/3TC/EFV drug regimen (2013 price)	145.47	145.47–280.72	[68]
Benefit 2: Increased Productivity			
Labor Force Participation:			
Average Monthly Min Wages (parameter 2.4 for LFP hours), USD (KES)	76.93 (6503)	57.42 to 103.60 (4854 to 8757)	[87]
Percentage rise in weekly hours worked after ARV treatment (LFP hours for parameter 2.3)	19%	3.7% to 34% (Normal Distribution, SE = 1.88, Mean = 4.6, Baseline total hours = 24.3)	[74]
Household Productivity:			
Increase in female hours spent collecting parameter in past week: (HP hours for parameter 2.3)			
Firewood	1.056	0.15 to 1.96 (Normal distribution, SE = 0.461)	[78]
Water	1.945	0.86 to 3.03 (Normal distribution, SE = 0.556)	[78]
Average Hourly Min Wage for House Worker (parameter 2.4 for HP hours), USD (KES)	0.85 (72)	0.58 to 1.03 (49–87)	[87]
Increase in ARV adherence due to increased abstinence:			
Hazardous Drinkers (number of patients non-adherent/exposed)	27.54% (19/69)		[7]
Non-Drinkers (number of patients non-adherent/exposed)	7.78% (112/1439)		[7]
Increase in the likelihood that an HIV+ patient which moves from non-abstinence to abstinence behavior will be ARV-adherent (parameter 2.2)	19.75%	14.75% to 24.75%	Calculated from above values
Currency Exchange (1USD: KES)	84.53		[61]

*Abbreviations: ARV* antiretroviral, *CBT* cognitive behavioral therapy, *CPI* consumer price index, *KNBS* Kenya National Bureau of Statistics, *3TC* lamivudine, *EFV* efavirenz, *GPRM* Global Price Reporting Mechanism, *KES* Kenyan shillings, *USD* US dollars, *SE* standard error

Range refers to the min-max interval used for sensitivity analysis

**Table 2:** Training costs

Details	Unit of issue	No. of units	Unit cost	Total KES per line item	Total KES per category	Total USD per line item	Total USD per category
Personnel							
Counselor supervisor consultants:							
2-week training plus 5 days training prep for 4 consultants	Day of pay × 4 consultants	76	KES 2,000	KES 152,000		$1,798	
Supervision after training (days = 12 sites × 18 weeks × 1 day/week)	Day of pay	216	KES 2,000	KES 432,000		$5,111	
Travel to sites (days = 12 sites × 18 weeks × 1 day/week)	Days of travel	216	KES 1,000	KES 216,000		$2,555	
Phone consulation after training (2 calls/site/mo for 8 mos)	Phone call	192	KES 300	KES 57,600		$681	
Counselor consultants total					KES 857,600		$10,146
Medical/Psychiatry trainers:							
10 day training of diploma nurses by 2 physicians (2–3 h session for each physician)	Days of training	10	KES 10,000	KES 100,000		$1,183	
Consultation after training, and training (10% FTE for 5 years for one physician)	Year salary	0.5	KES 1,440,000	KES 720,000		$8,518	
Psychiatry total					KES 820,000		$9,701
Per diems for trainees							
Counselors (12 days × 24 counselors)	Days of training × 24	288	KES 3,500	KES 1,008,000		$11,925	
Diploma Nurses (10 days × 12 nurses)	Days of training × 12	120	KES 3,500	KES 420,000		$4,969	
Per diems total					KES 1,428,000		$16,893
Training materials							
Training workbooks and treatment manuals	Piece	43	KES 900	KES 38,700		$458	
Audiorecorders	Piece	12	KES 4,000	KES 48,000		$568	
Rechargeable batteries	Piece	24	KES 200	KES 4,800		$57	
Battery charger	Piece	12	KES 1,040	KES 12,480		$148	
Posters	Piece	36	KES 1,100	KES 39,600		$468	
Sharp pointed pen	Packet	2	KES 500	KES 1,000		$12	
Whiteboard markers	Packet	2	KES 1,200	KES 2,400		$28	
Training materials total					KES 146,980		$1,739
Conference center							
Facility	Days of room rental	12	KES 12,000	KES 144,000		$1,704	
Lunch and tea (participants x days) ++	Lunches	486	KES 900	KES 437,400		$5,174	
Conference center total					KES 581,400		$6,878
Start up costs							
Furniture							
File cabinet	piece	12	16500	KES 198,000		$2,342	
Stacking plastic chair	piece	144	800	KES 115,200		$1,363	
Whiteboards	piece	12	8500	KES 102,000		$1,207	
Furniture total					KES 415,200		$4,912
Equipment							
Cellphone for calls for assessments	piece	12	4000	KES 48,000		$568	
Cashbox	piece	12	5999	KES 71,988		$852	
Equipment total					KES 119,988		$1,419
TOTAL TRAINING COSTS					KES 4,369,168		$51,688

Notes: These projections are based on 2-week training sessions at 12 sites

++ 24 counselors × 12 days = 288; 4 counselor trainers × 12 days = 48; 12 nurses × 10 days = 120; 3 physicians × 10 days = 30. Thus, total lunches = 486

Sources for salaries and per-diem rates:

Accommodation and subsistence allowance for officers travelling on duty within and outside Kenya. Memo from the office of the Prime Minister, Government of Kenya, 12 November 2009, Ref. No. MSPS 18/2A/(89)

Re-alignment of the salary structure for civil servants. Memo from the Office of the Prime Minister, Government of Kenya, 25 June 2012, Ref. No. MSPS 2/6/4A Vol. X/(2)

Re-alignment of teachers’ salary with those of civil servants. Memo from the Teacher Service Commission, 1 October 2012, Circular No 21/2012, Ref. TSC/ADM/192A/35

Rates of allowances payable to government sponsored trainees. Memo from the Office of the President, Government of Kenya, 1 November 2004, Ref. No. OP/CAB.2/12A

**Table 3:** Detailed scale-up costs per site

Details	Unit of Issue	No of units	Unit cost	Total KES per line item	Total KES per category	Total USD per line item	Total USD per category
Personnel							
Diploma Nurse	25% FTE	0.25	444,240	KES 111,060		$1,314	
Counselor	100% FTE	2	201,840	KES 403,680		$4,776	
Personnel total					KES 514,740		$6,089
Supplies							
Sharp pointed pen	pkt	2	500	KES 1,000		$12	
Whiteboard markers	pkt	2	1200	KES 2,400		$28	
Airtime for assessment coordinator^a^	75/week per office	1	20000	KES 20,000		$237	
Airtime for physician consultation	4 calls × 40 weeks	160	50	KES 8,000		$95	
Box files	pieces	5	155	KES 775		$9	
Suspension files	box	2	3500	KES 7,000		$83	
Photocopy	copy	2000	3	KES 6,000		$71	
Benzodiazepines (10% of pts per year)	patient	16	24	KES 384		$5	
Multivitamins	patient	16	16	KES 256		$3	
Supplies total					KES 45,815		$542
Provided by hospital facility:							
2 rooms							
Psychotropic medicines and benzodiazepines							
Participant Payments							
Participant payments (Participants × 6 visits)	Visits	960	200	KES 192,000		$2,271	
Participant payment total					KES 192,000		$2,271
TOTAL EXPENSES PER SITE PER YEAR					KES 752,555		$8,903

Notes: These projections are based on 1 year, 2 counselors, 1 location. This model can serve 160 (first year)-240 (years 2–5) participants (3 groups/week per counselor with 8 months of work.)

^a^500 KES/week × 40 weeks (assuming counselors will take leave at different times)

Sources for salaries and per-diem rates:

Accommodation and subsistence allowance for officers travelling on duty within and outside Kenya. Memo from the office of the Prime Minister, Government of Kenya, 12 November 2009, Ref. No. MSPS 18/2A/(89)

Re-alignment of the salary structure for civil servants. Memo from the Office of the Prime Minister, Government of Kenya, 25 June 2012, Ref. No. MSPS 2/6/4A Vol. X/(2)

Re-alignment of teachers’ salary with those of civil servants. Memo from the Teacher Service Commission, 1 October 2012, Circular No 21/2012, Ref. TSC/ADM/192A/35

Rates of allowances payable to government sponsored trainees. Memo from the Office of the President, Government of Kenya, 1 November 2004, Ref. No. OP/CAB.2/12A

Our program implementation model, based on field operations and expert consultation, contemplates a program that services 13,440 participants in 12 sites in Kenya across five years. In our base case, we assume the CBT treatment effect is maintained for two years, meaning that for every year which the intervention is rolled out, the participants of that year gain benefits associated with CBT for two years. Thus in our base case, we assume a 6-year time horizon. The program’s rollout was constructed from parameters based on our pilot experience in Eldoret, Kenya among 75 HIV-infected outpatients who reported hazardous or binge drinking, and our on-going randomized controlled efficacy trial with 614 randomized participants. The results, a full description of the pilot study, and an on-going trial are described elsewhere [47, 54, 59].

Methods for rollout were based on our previous work, with some modifications to enhance future sustainability [47, 54]. For example, while the pilot study was conducted in a large town (Eldoret) with a tertiary care medical center, our costs and methods for the rollout are based on delivering the intervention at the level of district or local hospital administered by the Ministry of Health. While paraprofessionals in our previous work possessed varying degrees of formal training from high school diploma to a bachelor’s degree, salaries and training in the rollout are based on high school diploma only, to make the intervention more realistic. Though we condensed the training period from 4 to 2 weeks in this exercise, we also extended the paraprofessional counselor consultation up to one year. Whereas a psychiatrist managed safety concerns (i.e., psychiatric risk and alcohol withdrawal symptoms) in our previous studies, our rollout methods are based on training diploma nurses to manage safety issues. Finally, while our past groups were delivered by gender-matched paraprofessionals, gender of the counselor in same-gender rollout group may not be always matched. Our rollout estimates are conservatively based on two counselors at each of 12 sites delivering two groups per week with eight same-gender participants per group, amounting to an annual workload of two counselors at each site would be 160 participants per site in year one. In years 2–5, the counselors are projected to have gained the training and experience to handle three groups per week, amounting to 320 participants per site. We have added monthly “maintenance” groups of post-CBT patients who would like to maintain or sharpen behavioral skills. We have reduced the rate of transport reimbursement per participant because of the rollout to more proximal location to participants, and we examine a wide range of transport reimbursement rates in our sensitivity analyses. Program evaluation methods would be left to each site and could be accomplished by providing a brief measure of alcohol use (e.g., AUDIT) before and after intervention participation.

### Inflation, exchange rate, discounting, and benefit-to-cost ratio

Benefits and costs were transformed into constant 2013 USD using the Kenyan consumer price index [60] and the average Kenyan shillings (KES) to U.S. dollar (USD) international exchange rate [61]. The value of all future benefits and costs were discounted at 3% per year. A benefit-to-cost ratio (BCR) was found by dividing the total discounted value of the benefits of CBT, by the total discounted value of the costs of CBT.

### Costs

Training costs in the first year included standard salaries in the communities, per diems for those attending trainings or traveling for work, and a housing allowance, with rates set by Kenyan government. Costs per site per year for the CBT rollout included personnel, furniture, equipment, supplies and participant transport reimbursement. Based on available resources at district and local hospitals, we assumed free provision of a space for the counselors, a group room, and access to limited psychotropic medications and benzodiazepines. In our rollout, counselors would be employed full-time, and diploma nurses part-time at 25%. For the base case scenario, participant transport reimbursement was set at 200 KES at each visit.

### Total and unit project costs

We estimated the total rollout project costs by adding the total training costs and the cost per site for 12 sites, discounted future costs (i.e., costs in years 2 to 5), and then divided by the number of projected participants across all 12 sites to obtain a per-participant unit cost.

### Benefits

We first measured the value of the economic benefits for a single outpatient over one year, then discounted this value over future years and summed the discounted benefits across all 13,440 CBT participants. This sum was subsequently employed as the numerator in our benefit-cost ratio.

### Benefit 1: lowered HIV incidence

Alcohol consumption has been shown to increase the spread of HIV infection [27, 37, 62–65]. A recent study by Braithwaite et al. showed that alcohol use was responsible for an estimated 13% of new HIV infections; specifically citing our task-shifted pilot model and estimating that it could prevent nearly half of these new HIV infections caused by alcohol use [36]. Thus by lowering alcohol consumption, the CBT intervention would avert a large percentage of new HIV infections, and could in turn create future savings in terms of averted medically-related costs.

We modeled the annual savings accrued through averted costs from reduced likelihood of HIV transmission per HIV-negative patient as the multiplication product of:

1. The likelihood that a CBT-treated patient will exhibit abstinence for a year (parameter 1.1)
2. The reduced likelihood that an HIV-negative patient will be infected given a single HIV+ patient within the CBT treatment population abstains from alcohol (parameter 1.2)
3. The cost of treating a case of HIV for a year (from a provider’s perspective), (parameter 1.3)

The parameter 1.1 was taken from a randomized control trial done by Papas et al. [54]. This study found that 69% of CBT participants reported abstinence after a 90 day follow-up, and 38% of the usual care control group reported abstinence at follow-up. Parameter 1.1 was found by subtracting the difference between the treatment group and the control group. The parameter 1.2 was found from a simulation model of HIV disease progression and transmission by Braithwaite et al. [36]. Through a 135-article review, Braithwaite et al. identified and modeled three consequences of alcohol-use which increase the incidence of HIV transmission: increased risk of condom non-use, increased risk of ARV non-adherence, and increased STI prevalence. Braithwaite et al.’s model estimated 13% of new HIV infections in Kenya were attributable to alcohol use.

Parameter 1.3 was found by summing the costs of anti-retroviral drugs (ARVs), clinic visits, lab tests, clinic support services, and hospital fixed costs (costs related to cleaners, hospital equipment, etc.). All costs except the cost of ARVs were taken from a cross-sectional survey by Larson et al., which looked at patient level cost data across three different clinics in Kenya [66]. The World Health Organization (WHO) recommends tenofovir/3TC/EFV as a first-line ARV treatment [67], and the annual cost of tenofovir/3TC/EFV in Kenya was found using the WHO’s Global Price Reporting Mechanism [68].

### Benefit 2: increased labor force and household productivity

Studies have shown that alcohol consumption is positively correlated with ARV non-adherence; and conversely, that lower levels of alcohol consumption and abstinence are associated with better ARV adherence [7, 69, 70]. CBT—in reducing alcohol consumption—has been identified as a method to increased ARV adherence [71–73]. ARV adherence and its resulting medical and functional benefits, in turn, have been shown to increase labor force participation (LFP) [74–77] and increase household productivity (HP) [78]. Thus, through a mechanism of ARV adherence, CBT can increase LFP and HP. We estimated the monetary benefits of LFP and HP as per person-year values. These monetary benefits were calculated as the multiplicative product of:

1. The likelihood that a CBT-treated patient will exhibit abstinence for a year (same value as in benefit 1, i.e. parameter 1.1). Note that we do not call this parameter 2.1, but parameter 1.1. We also do not name any variables in this paper as parameter 2.1 in order to avoid confusion.
2. The increase in the likelihood that an HIV+ patient who moves from non-abstinence to abstinence behavior will be ARV-adherent. (Parameter 2.2)
3. The additional ARV-related hours worked annually per person from increased LFP/HP (the additional hours for LFP were used to calculate the value of the benefits for LFP, and the additional hours for HP were used calculate the value of the benefits for HP). (Parameter 2.3)
4. The corresponding wage value for the hours measured in parameter 2.3. (Parameter 2.4)

A major challenge in estimating parameters 2.2 and 2.3 was accounting for potential patient differences in ARV-adherence and ARV-initiation respectively. In estimating parameter 2.3, in order to take into account different levels of ARV-adherence, multiple levels of ARV-adherence would need to measured and tracked within a Kenyan population. Such data was not available. Patient differences in ARV-treatment initiation times, meanwhile, can potentially bias measurements of parameter 2.3. Those who initiated ARV-treatment before CBT-treatment are likely to exhibit lower positive benefits from increases in ARV-adherence than those who initiated ARVs during CBT-treatment (and would move from no ARV-treatment to full ARV-adherence post-CBT treatment).

We address problems associated with different levels in ARV-adherence in our estimation of parameter 2.2 by looking only at the effect of full ARV-adherence, and categorizing all other levels of adherence as non-adherence. This binary indicator implies that we need only find the effect of abstinence on increasing the likelihood that a patient moves from any given level of ARV-adherence below full adherence (i.e. non-adherence) to full adherence, rather than tracking multiple movements across multiple levels of ARV-adherence. Thus parameter 2.2 was found from a cross-sectional survey of 2920 ARV-treated patients from clinics in Cote d’Ivoire, Benin, and Mali by Jaquet et al. [7]. To estimate parameter 2.2, we subtracted the fraction of non-drinkers which were non-adherent from the fraction of hazardous drinkers which were non-adherent.

To deal with potential ARV-initiation-related bias in the estimation of Parameter 2.3, we used results from the individual fixed-effects regressions of studies which measured the effects of ARV-adherence on random samples of Kenyan patients [74, 78]. By measuring the effect of ARV-adherence on productivity in a random sample of patients, the ARV-initiation times of the studied population likely resemble our modeled CBT treatment population, and thus the measured effect used in our input of Parameter 2.3 already takes into account the differences in ARV-initiation of our modeled population. We use results from a study by Thirumurthy et al. to measure the LFP hours of parameter 2.3, using coefficients from their regression of LFP on consistent 6-month ARV-adherence [74]. The value of the per person-year values of the gains in LFP was counted for both men and women, because the regression coefficients were found using control groups comprised of patients from randomly selected households from Kenyan census data and the treatment groups comprised of patients from randomly selected households from the Mosoriot health clinic in Kenya. This random selection ensures that although men are more likely to be engaged in labor market activities than women [74], the results take this into account and can be applied across men and women.

The value of the per person-year values of the gains in HP, on the other hand, was counted only for women, as the regression coefficients from which the HP hours of parameter 2.3 was estimated were specific to female household productivity [78]. We assumed that half of our simulated population was female.

The LFP value for parameter 2.4 was found by taking the mean across all monthly minimum wage listings from the Kenyan Ministry of Labor’s 2013 Regulation of Wages, and multiplying this number by 12 to yield an annual estimate. Similarly, the HP value for parameter 2.4 was found by taking the mean hourly wage for a house worker across all geographic locations.

### Statistical and sensitivity analyses

To characterize and take into account the potential error of the studies used for the parameters of our base case, we first conducted one-way sensitivity analyses where we changed all inputs, one at a time, across a range of values. The value of the bounds of this range was found by taking the 95% confidence interval (CI) around the base case values; or if the CI was unavailable, the absolute range of values for which the input was observed. In the cases of the cost of CBT rollout per participant, parameter 1.1 and parameter 2.2, literature values for ranges were not available, so we chose a broad range of values around the base case to reasonably test robustness. In addition to the inputs listed in Table 1, we also varied the treatment effect length, the discount rate, and participant transport reimbursement.

Additionally, to further characterize the inherent uncertainty all inputs in Table 1 were simultaneously varied in a Monte Carlo simulation where, for each cohort of participants, data was drawn from the same ranges as in the one-way sensitivity analysis. Simulations assumed a logarithmic (right-skewed) distribution for costs, a normal distribution for inputs whose ranges were CIs (with a standard deviation equal to the standard error used to calculate the CI), and uniform distribution for all other ranges. We ran Monte Carlo simulations with 10,000 replications for each independent cohort of 13,440 participants using multiprocessor Stata 14 [79]. All simulations assumed the base case 3% discount rate, and the treatment effect length was set to either one, two, three, four, five, or ten years for each set of 10,000 replications. All sets of replications for each treatment effect length are listed in Fig. 3. For both univariate and probabilistic sensitivity analyses, when the treatment effect length was adjusted, the time horizon was also adjusted as well to take into account the final group’s benefits across the treatment effect length.

## Results

**Fig. 1:**
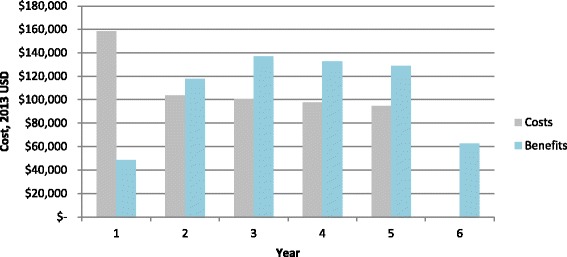
Costs and potential benefits accrued (in time-discounted 2013 USD). Figure 1 illustrates the monetary costs and potential benefits from a cognitive behavioral therapy which would be used to reduce alcohol abuse among 13,440 persons living with HIV in Kenya. Values of cost and benefits were discounted at a rate of 3% assuming that they occur at the end of each year, thus costs in year 1 were not discounted

The overall benefit-cost ratio was 1.13. Figure 1 summarizes the costs and benefits for the base case CBT implementation (costs are in the first bar labeled in gray, benefits are shown in the second bar labeled in blue). Training costs in the first year were $158,000, which were higher than subsequent years, primarily due to personnel costs. However, costs declined across the final four years to about $94,000 in year 5. The total discounted cost over the five-year rollout was approximately $554,000. Over the five year period, the average cost-per-participant was $44. (Cost breakdowns are presented in Tables 2 and 3). Benefits totaled $49,000; $118,000; $137,000; $133,000; $129,000; and $62,000 in years 1, 2, 3, 4, 5, and 6 respectively. Note that benefits continued in year 6, despite the costs of the program ending in year 5. The average annual savings from decreased HIV incidence (benefit 1) was $11 per patient; and the annual value for increased productivity (benefit 2) was $14.50 per patient. The total value of benefit 1 and benefit 2 was estimated to be approximately $628,000.

**Fig. 2:**
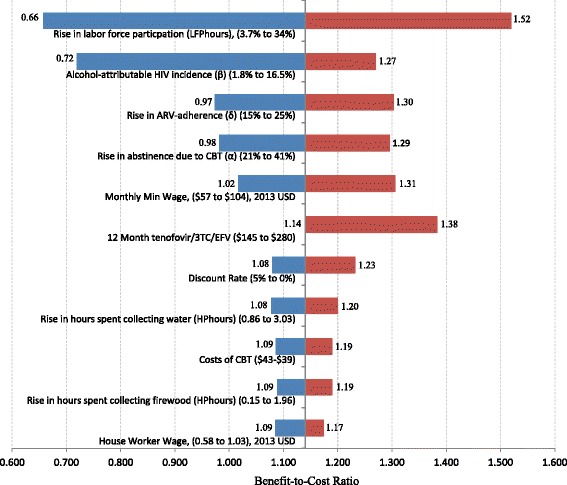
One-way sensitivity analyses for input variables that most affect the base case results. In the figure above, “Rise in labor force participation” is a measure of the percentage rise in weekly hours worked after ARV treatment; “Alcohol-attributable HIV incidence” is the percentage of HIV incidence attributable to alcohol consumption; “Rise in ARV-adherence” is the Increase in the likelihood that an HIV+ patient which moves from non-abstinence to abstinence behavior will be ARV-adherent; “Rise in abstinence due to CBT” is the Difference between intervention and usual care in percentage of patients reporting abstinence at 90 day follow-up; “Monthly Min Wage” is a measure of the Average Monthly Minimum Wage in Kenya in USD; “12 Month tenofovir/3TC/EFV” is the cost of a 12 Month tenofovir/3TC/EFV drug regimen expressed in 2013 USD; “Rise in hours collecting water” is the increase in female hours spent collecting water in past week; “Costs of CBT” is the cost of the CBT rollout per participant; “Rise in hours spent collecting firewood” is the increase in female hours spent collecting firewood in past week; “House worker min wage” is the Average Hourly Minimum Wage for House Worker. The numbers in the parentheses represent the upper and lower bounds of the sensitivity analysis. The numbers listed at the left and right hand side of the bars represent the benefit to cost ratio which would result from the target variable taking on the corresponding max or min value. Note that the vertical axis is at 1.13, but that all cost-benefit ratios above 1.0 are cost-saving and thus most variables maintain that CBT is cost saving across the entire range of variables. Ratios rounded to nearest hundredth. Abbreviations: BCR, benefit-to-cost ratio; CBT, cognitive behavioral therapy. The vertical axis intersects the horizontal axis at approximately 1.13

Figure 2 shows the input variables that most affected the base case scenario in one-way sensitivity analyses. For nearly all inputs shown in Fig. 2, CBT remained cost-savings with a benefit-cost ratio greater than 1. Not pictured in Fig. 2 are values associated with non-drug related inputs associated with parameter 1.3. These four inputs had little effect on the benefit-cost ratio, and when varied across their ranges, yielded benefit-cost ratio ranges of: 1.138 to 1.140 (support services); 1.13 to 1.15 (lab tests); 1.13 to 1.15 (visits); and 1.11 to 1.18 (fixed costs). Also not shown in Fig. 2 is the uncertainty associated with the assumed treatment effect length. When we adjusted our assumption to the treatment effect lasting 1, 5 and 10 years, the benefit-cost ratio changed to 0.58; 2.72; and 5.05 respectively.

**Fig. 3:**
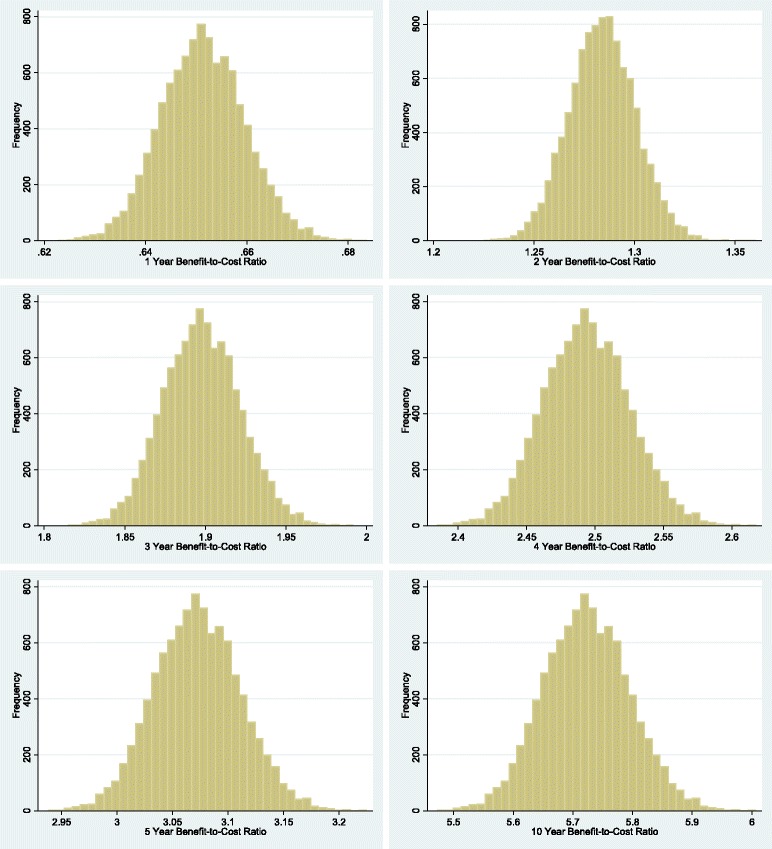
Distribution of benefit-to-cost ratios from Monte Carlo simulations. Figure 3 shows the results of our probabilistic sensitivity analysis with discounted net benefits. Separate sets of simulations were independently run for the assumption that program effect duration was 1, 2, 4, 5, or 10 years. Each set of simulations was made up of 10000 repetitions done across an independent sample of 13440 participants. Each set of simulations is also shown with a separate graph

In Fig. 3, we further characterize the inherent uncertainty in the parameters by summarizing the probabilistic sensitivity analyses. The results were split into groups based on treatment effect duration; for each group we present separate Monte Carlo simulations with 10,000 repetitions, each with an independent sample of 13,440 participants. The mean benefit-cost ratio under assumptions that the CBT treatment effect lasted for 1, 2, 3, 4, 5 or 10 years was 0.65 (95% CI 0.64–0.66); 1.28 (95% CI: 1.26–1.31); 1.90 (95% CI 1.86–1.94); 2.49 (95% CI 2.44–2.55); 3.07 (95% CI 3.01–3.14); and 5.72 (95% CI: 5.61–5.84) respectively.

## Discussion

Previous research—which used only health outcomes— showed that CBT was generally cost-effective, but only cost-saving under strict assumptions (i.e., program costs of less than $1 per individual) [36, 37]. In this study, across a broad range of assumptions, task-shifted CBT was not only favorable, but even cost-saving from a societal perspective, when we included health *and* economic productivity benefits. Our base-case scenario showed a benefit-cost ratio of 1.13 when the treatment effects were assumed to last two years; and the benefit-cost ratio increased to 1.90 with a 3-year treatment maintenance scenario.

Our results are consistent with a related exercise which characterized task-shifted CBT for alcohol use reduction in Kenya as cost-effective [37]. However, the extant results go further, showing not only reductions in morbidity and mortality associated with the scale-up, but also monetary cost-savings for the system as a whole in Kenya. Some costs included in our analyses such as specific per-diem rates and housing allowances, are set by the Kenyan government. These are likely not required in other sub-Saharan African countries, so the benefit-cost ratio would likely be higher in those settings. The provision of transport reimbursement increases the cost of the intervention, but likely enhances the effectiveness of the intervention by facilitating better attendance.

A strength of this study is that the scale-up was constructed using in-depth knowledge and data of the training and implementation of CBT procedures from two clinical trials. The costing exercise presents a novel and realistic way of shifting the CBT tasks to paraprofessionals in an organized, feasible and scalable manner. The training, enrollment and compensation procedures have been implemented in our past and current trials. Furthermore, the potential economic benefits are based on rigorous analyses based in Kenya with the same or similar populations [74, 78], and have been recognized to be more widely applicable [80]. The effect of this approach could support the country’s 2010 Alcoholic Drinks Control Act which seeks to tighten legislation against alcohol abuse while also, for the first time, ensuring the right to access to treatment programs [81].

The input parameter most affecting the results was the duration of CBT-alcohol reduction treatment effects. It was difficult to choose a base case scenario because evidence regarding the long-term effect length of CBT for alcohol-use reduction is limited. One reason may be because the length of follow-up of CBT studies is often constrained by the time limits of grant cycles. Despite these limitations, there is reason to believe that CBT treatment effects could be maintained over a long period without major reductions in effectiveness. In a 128-person treatment sample, Kadden and colleagues observed only marginal reductions in treatment effects in alcohol use at 18-month post-treatment follow up; including a group CBT condition [82, 83]. Although we felt a base case of a two-year effect was reasonable, given the uncertainty surrounding the treatment effects, we also conducted sensitivity analyses where the base case treatment effect was adjusted to 1, 5, and 10 years. Our sensitivity analysis showed that our base case results held for treatment effect lengths greater than two years, but at a one-year duration assumption, CBT did not achieve cost-neutrality. This exercise in modeling reveals that while a task-shifted CBT has excellent potential for considerable cost-savings, if treatment effects are particularly short, it is unlikely that CBT will be cost-savings. It may still be highly cost-effective (i.e., averting a DALY for less than the per capita GDP), but it may not save money to the government in the long run. Again, very few interventions achieve the status of cost saving [84, 85].

This study has limitations. First, the CBT effects are based on a small pilot study. Thus, we are currently conducting a larger trial, and expect to update the results presented here in future research. Second, due to the limited information regarding the effects of various levels of adherence, we have dichotomized the treatment effect of our modeled CBT as “abstinent” vs. “non-abstinent. It is possible that if continuous data were available regarding the effect of reducing drinking by one drink, then our modeled CBT would be shown to have even greater levels of benefits in terms of ARV adherence and reduced HIV infectivity given the positively correlation between alcohol use and these modeled benefits [7, 31, 36, 37, 64–67, 71–73]. Third, cost-benefit models regarding future cost-savings carry inherent uncertainty as assumptions must be made regarding the consequences of an intervention. As discussed above, the input parameter most affecting CBT treatment cost-neutrality was the CBT treatment effect maintenance. However, to deal with the uncertainty of this and all other variables, we conducted various sensitivity analyses: assumptions regarding the parameters in this model were varied one at a time, as well as simultaneously across ranges presented in the literature.

## Conclusion

A scaled-up task-shifted CBT intervention to reduce alcohol use among HIV+ persons receiving ARVs can be not only cost-effective but also potentially cost-saving in settings such as Kenya, with generalized HIV epidemics and high rates of alcohol use. This analysis can be helpful for other countries planning to use task-shifting of CBT to reduce alcohol use, not only to reduce morbidity and mortality related to HIV disease, but also to generate potential economic gains. In particular, the study demonstrates how *ex ante* economic evaluation results may be generated such that countries can evaluate the effectiveness of other mental and public health approaches to improving ARV adherence, reducing risk sexual behaviors, and reducing HIV incidence.

## Abbreviations

3TC: Lamivudine
AIDS: Acquired immune deficiency syndrome
ARV: Antiretroviral
AUD: Alcohol use disorder
BCR: Benefit-cost ratio
CBA: Cost-benefit analysis
CBT: Cognitive behavioral therapy
CI: Confidence interval
DALY: Disability-adjusted life year
EFV: Efavirenz
GDP: Gross domestic product
HIV: Human immunodeficiency virus
HP: Household productivity
KES: Kenyan shilling
LFP: Labor force participation
MAT: Medication assisted therapy
USD: United States dollar
WHO: World Health Organization
